# Supplementary material for: A UMLS-based spell checker for natural language processing in vaccine safety
Source: BMC Med Inform Decis Mak. 2007 Feb 12;7:3. doi: 10.1186/1472-6947-7-3 (PMC1805499; doi:10.1186/1472-6947-7-3)
Supplement: Additional file 13 — RAP application directory. Sets up directory for RAP (RDF application for PHP) source code [file 1472-6947-7-3-S13.gz › rap/doc/phpdoc/__filesource/fsource_rdql__rdqlRdqlEngine.php.html]

xml version="1.0" encoding="iso-8859-1"?


File Source for RdqlEngine.php


# Source for file RdqlEngine.php

Documentation is available at RdqlEngine.php

```
1. <?php

3. // ----------------------------------------------------------------------------------
4. // Class: RdqlEngine
5. // ----------------------------------------------------------------------------------


9. /**
10. * Some general methods common for RdqlMemEngine and RdqlDbEngine
11. *
12. * <BR><BR>History:<UL>
13. * <LI>07-27-2003                : First version of this class</LI>
14. *
15. * @version  V0.9.1
16. * @author   Radoslaw Oldakowski <radol@gmx.de>
17. *
18. * @package rdql
19. * @access public
20. */

22. Class RdqlEngine extends Object{


26. /**
27. * Prints a query result as HTML table.
28. * You can change the colors in the configuration file.
29. *
30. * @param array $queryResult [][?VARNAME] = object Node
31. * @access private
32. */
33. function writeQueryResultAsHtmlTable($queryResult) {
34. // Import Package Utility
35. include_once(RDFAPI_INCLUDE_DIR.PACKAGE_UTILITY);

37. if (current($queryResult[0]) == NULL) {
38. echo 'no match<br>';
39. return;
40. }

42. echo '<table border="1" cellpadding="3" cellspacing="0"><tr><td><b>No.</b></td>';
43. foreach ($queryResult[0] as $varName => $value)
44. echo "<td align='center'><b>$varName</b></td>";
45. echo '</tr>';

47. foreach ($queryResult as $n => $var) {


50. echo '<tr><td width="20" align="right">' .($n + 1) .'.</td>';
51. foreach ($var as $varName => $value) {
52. echo INDENTATION . INDENTATION . '<td bgcolor="';
53. echo RDFUtil::chooseColor($value);
54. echo '">';
55. echo '<p>';

57. $lang  = NULL;
58. $dtype = NULL;
59. if (is_a($value, 'Literal')) {
60. if ($value->getLanguage() != NULL)
61. $lang = ' <b>(xml:lang="' . $value->getLanguage() . '") </b> ';
62. if ($value->getDatatype() != NULL)
63. $dtype = ' <b>(rdf:datatype="' . $value->getDatatype() . '") </b> ';
64. }
65. echo  RDFUtil::getNodeTypeName($value) .$value->getLabel() . $lang . $dtype .'</p>';
66. }
67. echo '</tr>';
68. }
69. echo '</table>';
70. }

72. } // end: Class RdqlEngine

74. ?>
```

Documentation generated on Thu, 7 Jul 2005 13:43:13 +0200 by phpDocumentor 1.3.0RC3
